# Role of toll-like receptor 4 on the immune escape of human oral squamous cell carcinoma and resistance of cisplatin-induced apoptosis

**DOI:** 10.1186/1476-4598-11-33

**Published:** 2012-05-14

**Authors:** Zujun Sun, Qingqiong Luo, Dongxia Ye, Wantao Chen, Fuxiang Chen

**Affiliations:** 1 Department of Clinical Laboratories, Ninth People’s Hospital Affiliated to Shanghai JiaoTong University School of Medicine, Shanghai 200011, China; 2 Department of Oral and Maxillofacial Surgery, Ninth People’s Hospital, Shanghai JiaoTong University School of Medicine, Shanghai, China

**Keywords:** Toll-like receptors, Oral squamous cell carcinoma, Lipopolysaccharide, Myeloid differentiation primary response gene 88

## Abstract

**Background:**

Toll-like receptor 4 (TLR4) is expressed on immune cells as a sensor that recognizes lipopolysaccharide (LPS), a microbial conserved component. It has recently been determined that the expression of TLR4 is also found in various types of tumor cells. Cisplatin is a widely used chemotherapeutic agent for oral squamous cell carcinoma (OSCC) treatment. However, the mechanisms responsible for cisplatin resistance are not well understood.

**Results:**

The present study was designed to elucidate the role of TLR4 expression in human OSCC regarding immune escape and apoptotic resistance to cisplatin. TLR4 and the myeloid differentiation primary response gene 88 (MyD88) were highly expressed in OSCC cell lines. Upon LPS stimulation both NF-κB and p38 MAPK pathways were activated in OSCC cell lines, followed by the production of large quantities of IL-6, IL-8 and VEGF compared with human immortalized oral epithelia cells (HIOECs). OSCC cell lines were found to be resistant to cisplatin-mediated apoptosis after pretreatment with LPS.

**Conclusions:**

Our results suggested that TLR4 was functionally expressed in human OSCC cells and development of resistance to cisplatin in human OSCC might occur through the mechanism involving TLR4 and its signaling pathway. Suppression of TLR4 and its signaling pathway might thus elevate sensitivity to cisplatin and potentially help improve the prognosis of patients with OSCC.

## Background

The prognosis of human oral squamous cell carcinoma (OSCC) is usually poor with a 5-year survival rate of approximately 50 ~ 60%, which has generally been attributed to the insensitivity of most patients to chemotherapy [1,2]. Recently, there has been a growing recognition of interest in anti-tumor functions initiated by the innate immune response. The role of toll-like receptors (TLRs) and their signaling in tumor immune escape and resistance to apoptosis, for example, is among the frontiers of exploration [3,4].

TLRs are type I transmembrane proteins with extracellular domains comprised largely of leucine-rich repeats and intracellular signaling domains that play a crucial role in inflammation and host defense against invading microorganisms through the recognition of pathogen-associated molecular patterns (PAMPs) such as LPS, lipopeptides, dsRNA, and bacterial DNA [4]. After binding with ligands, TLRs and Toll/interleukin-1 receptor-like domain (TIR) are dimerized and undergo a conformational change that is required to recruit downstream signaling molecules, such as MyD88. In mammals, the TLR family consists of at least 12 members expressed predominantly on the surface of macrophages and various immune cells [5]. LPS is specifically recognized by TLR4 [6,7]. More recent studies have demonstrated TLRs expression in a broad variety of tumor tissues and tumor cell lines [8-11]. Their activated signaling pathways in cancer cells could have profound consequences for tumor growth by promoting cancer progression, anti-apoptotic activity, and resistance to host immune responses [12]. Although there have been reports of TLRs in various tumor types, the function of these receptors in OSCC prognosis and resistance to chemotherapy remains unknown [13].

OSCC is sensitive to cisplatin-based chemotherapy in patients with an initial response to treatment. However, chemoresistance is observed in the treatment of recurrent patients because of acquired or intrinsic resistance. The main mechanisms and pathways that lead to clinical resistance to cisplatin in OSCC have not been well defined. So far, little is known about the function and biological importance of TLR4 on OSCC chemoresistance. Thus, it is important to address the pattern of TLR4 expression on OSCC cell lines and to determine the pathophysiological significance of TLR4 signaling in the immune escape and chemoresistance of OSCC. The purpose of this study was to explore the expression and role of TLR4 in OSCC and how TLR4 signaling correlated with cisplatin resistance in OSCC.

## Results

### Expression of TLR4 and MyD88 in human OSCC tissues and cells

**Figure 1:**
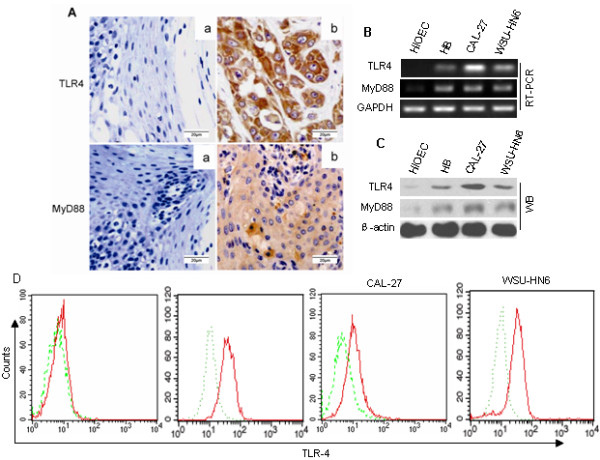
**Expression of TLR4 in human OSCC tissues and cell lines. *A. *** Immunohistochemical examination of the expression of TLR4 and MyD88 in OSCC tissues and adjacent non-malignant epithelia. (a) adjacent non-malignant epithelia; (b) OSCC tissue (magnification × 400). ***B-D.*** TLR4 and MyD88 in HIOEC and OSCC cell lines were analyzed by RT-PCR *(****B),*** Western blot *(****C)*** and flow cytometry (***D)***. Green line-covered regions represent isotype controls and red regions represent the detection of TLR4 with mAb.

To investigate whether TLR4 and its adaptor molecule MyD88 were expressed in human OSCC tissues, 30 human OSCC specimens and 10 normal oral tissue samples adjacent to the tumor were assessed *in situ* in paraffin sections. In tumor samples, TLR4 was found to localize to the cell membrane and cell cytoplasm and MyD88 localized to the cell cytoplasm only. We also detected the expression of TLR4 and MyD88 in the normal mucosa adjacent to the tumor, but the expression was quite weak (Figure 1A). The expression levels of TLR4 and MyD88 *in situ* were correlated with tumor differentiation. TLR4 was highly expressed in well-differentiated and moderately-differentiated tumors, but weakly expressed in poorly differentiated tumors (p = 0.018, Table 1). The expression pattern of MyD88 in OSCC tumor tissues was similar to that of TLR4 (p = 0.034, Table 1). However, TLR4 and MyD88 expression were not correlated with other parameters such as tumor stages, nodal status, tumor size or tumor site. Additionally, we detected up-regulated TLR4 and MyD88 mRNA in three OSCC cell lines-HB, CAL-27 and WSU-HN6, compared with HIOEC by RT-PCR as shown in Figure 1B. Western blot analysis also revealed high expression of TLR4 and MyD88 in OSCC cell lines (Figure 1C). Consistent with RT-PCR and Western blot results, FACS analysis demonstrated that TLR4 expression in OSCC cell lines was quite high, while the expression of TLR4 in HIOEC was very low (Figure 1D). Thus, the different expression levels of TLR4 between HIOEC and OSCC cell lines suggest that TLR4 may be functionally important in human OSCC cells.

**Table 1:** Correlation between the clinicopathological features and the expressions of TLR4 and MyD88

***Characteristics***	***Case no.***	***TLR4 positive grade***	***Nonparametric tests value***	***P.value***	***MyD88 positive grade***	***Nonparametric tests value***	***P.value***
Tobacco							
Yes	8	1.50 ± 0.92	Z = -0.101	0.919	1.37 ± 0.74	Z = -0.565	0.572
No	22	1.45 ± 0.74			1.59 ± 0.73		
Alcohol							
Yes	2	2.00 ± 1.41	Z = -0.702	0.472	2.00 ± 1.00	Z = -1.092	0.275
No	28	1.43 ± 1.74			1.5 ± 0.74		
Sex							
Male	22	1.59 ± 0.73	Z = -1.294	0.196	1.36 ± 0.72	Z = -0.396	0.696
Female	8	1.12 ± 0.83			1.75 ± 1.04		
Tumor site							
Oral cavity	20	1.55 ± 0.82	χ2=1.635 d.f. = 3	0.652	1.45 ± 0.64	χ2=0.032 d.f. = 3	0.999
Gingiva	4	1.25 ± 0.95			1.5 ± 1.29		
Mouth floor	3	1.33 ± 0.57			1.33 ± 1.15		
Other	3	1.33 ± 0.57			1.66 ± 1.15		
Tumor stage							
T1	3	1.66 ± 1.15	χ2=1.698 d.f = 3	0.637	1.66 ± 0.57	χ2=0.498 d.f. = 3	0.919
T2	14	1.57 ± 0.75			1.35 ± 0.74		
T3	6	1.50 ± 0.83			1.50 ± 1.04		
T4	7	1.14 ± 0.69			1.57 ± 0.97		
Nodal status							
N0	14	1.57 ± 0.85	Z = -0.495	0.621	1.50 ± 0.65	Z = -0.224	0.823
N1	16	1.37 ± 0.71			1.43 ± 0.96		
Pathological differentiation grade							
Well	13	1.76 ± 0.72	χ2=8.014 d.f = 2	0.018	1.61 ± 0.76	χ2=6.774 d.f. = 2	0.034
Moderatedly	13	1.61 ± 0.50			1.69 ± 0.75		
poorly	4	0.50 ± 0.57			0.50 ± 0.57		

### LPS promotes IL-6, IL-8 and VEGF production via ligation of TLR4 in human OSCC cells

**Figure 2:**
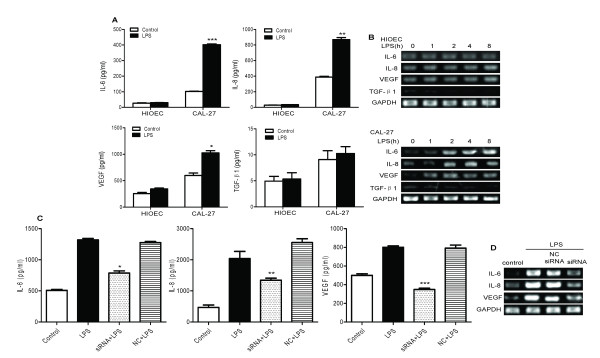
**LPS increased the expression and secretion of IL-6, IL-8 and VEGF through TLR4 in a human OSCC cell line instead of HIOEC cells. *A. *** HIOEC and CAL-27 cells (5 × 10^5^ ml^-1^) were stimulated with LPS (1 μg/ml) for 24 h, and then cytokines and chemokines in the supernatants were assayed using sandwich ELISA. Protein levels (pg/ml) are expressed as mean ± SD in three independent experiments (***p < 0.001, **p < 0.01, *p < 0.05, compared with control). ***B.*** RT-PCR analysis of IL-6, IL-8, and VEGF and TGF-β mRNA expression in HIOEC and CA-27 cells stimulated with LPS (1 μg/ml) for indicated time points. ***C.*** To determine the cytokine levels, supernatants were collected from cultured tumor cells after stimulation with LPS for 24 h with or without TLR4 siRNA transfection. Results are expressed as mean ± SD of cytokine concentrations (pg/ml) in three independent experiments (***p < 0.001, **p < 0.01, *p < 0.05, compared with the NC siRNA group). ***D.*** LPS-induced expression of IL-6, IL-8, and VEGF was inhibited by TLR4 siRNA in CAL-27 cells as determined by RT-PCR.

The cytokines secreted by tumor cells can promote immune suppression and angiogenesis, which in turn eventually facilitate tumor survival and metastasis. To investigate the physiological and pathological roles of TLR4 in OSCC cells, we treated HIOEC and CAL-27 cells with LPS for 24 h, and then collected the supernatants in order to determine the levels of IL-6, IL-8, VEGF and TGF-β by ELISA. We found that upon LPS stimulation, CAL-27 cells produced large quantities of IL-6, IL-8 and VEGF, but the level of TGF-β was not significantly changed (Figure 2A). In order to verify the results obtained by ELISA, we performed RT-PCR and detected up-regulated mRNA expression for IL-6, IL-8, and VEGF in CAL-27 cells after LPS stimulation (Figure 2B). No elevated mRNA expression or secretion of IL-6, IL-8 and VEGF were observed in HIOEC cells after LPS stimulation (Figure 2A and B). In order to verify the necessity of TLR4 signaling to achieve production and expression of IL-6, IL-8 and VEGF in human OSCC cell lines induced by LPS, we tested to see whether TLR4 siRNA could block the production of these cytokines. TLR4 siRNA was transfected into CAL-27 cells, followed by stimulation with LPS, and the result was that the production and expression of IL-6, IL-8 and VEGF in CAL-27 cells were nearly abolished (Figure 2C and D). Similar results were obtained for HB and WSU-HN6 cells (Additional file 1: Figure S1).

### LPS activates p38 MAPK and NF-κB signal pathways through TLR4 in OSCC cells

**Figure 3:**
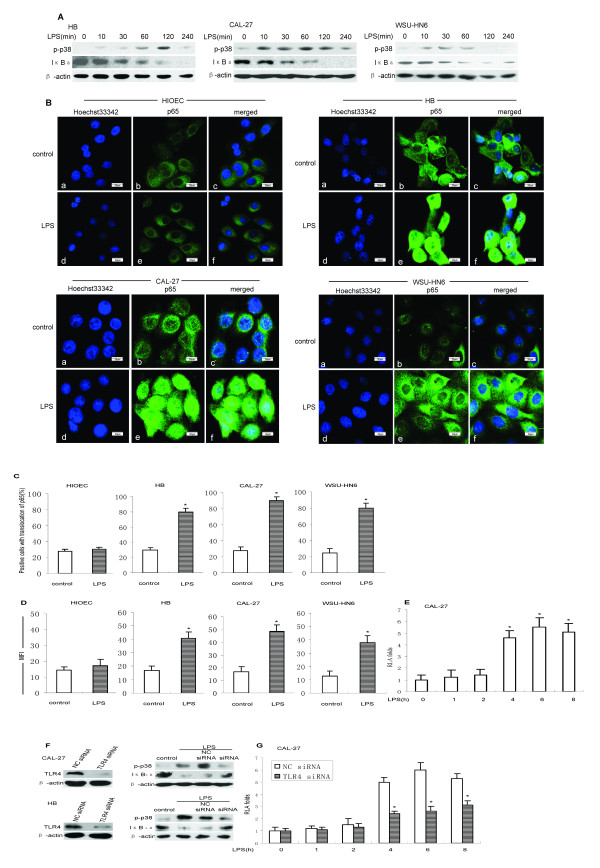
**LPS induced activation of p38 MAPK and NF-κB pathways through TLR4 in OSCC cell lines. *A. *** LPS induced p38 MAPK phosphorylation and I-κBa degeneration for indicated time points in OSCC cell lines. ***B.*** HIOEC and OSCC cells plated overnight were stimulated with LPS (1 μg/ml) for 8 h and then stained as described in Materials and Methods. a to c, control cells were untreated; a and d, nuclei are stained blue; b and e, the p65 subunit of NF-κB is stained green; c, an overlay of a and b; f, an overlay of d and e. ***C.*** Mean ± SD of the percentage of cells with p65 translocation. 200 cells were randomly counted. Results are representative of three independent experiments performed with each cell line (*p < 0.05, compared with control). ***D.*** Mean fluorescence intensity (MFI) of nuclear p65 expression was determined in OSCC cell lines. Results are expressed as mean ± SD of MFI for three independent experiments performed with each cell line (*p < 0.05, compared with control). ***E.*** Determination of the LPS-activated, NF-κB-dependent transcriptional activity in CAL-27 cells. The cells were transiently cotransfected with pNF-κB-Luc and pRenilla. Then after 24 h, cells were either left untreated or stimulated with 1 μg/ml of LPS for various times. Luciferase activity was assessed in the cells (*p < 0.05, compared with control). ***F.*** TLR4 was effectively silenced as determined by Western blot and TLR4 siRNA suppressed the activation of p38 MAPK and NF-κB pathways in OSCC cell lines treated with LPS. ***G.*** NF-κB-dependent transcriptional activity was inhibited by TLR4 siRNA in CAL-27 cells. CAL-27 cells were transiently transfected with NC siRNA and TLR4 siRNA, then cotransfected with pNF-κB-Luc and pRenilla. 24 h later, the cells were either left untreated or stimulated with 1 μg/ml of LPS for various time. Luciferase activity was assessed in the cells stimulated with or without LPS. (*p < 0.05, compared with NC siRNA).

LPS-triggered TLR4 signaling usually involves two different pathways: the p38 mitogen-activated protein kinase (MAPK) signaling pathway and the NF-κB signaling pathway. To determine whether LPS could activate p38 MAPK and NF-κB pathways in OSCC cell lines, we treated OSCC cell lines with LPS for various time periods followed by protein extraction from the cells. Western blot analysis showed that p38 MAPK phosphorylation was induced by LPS in OSCC cell lines as early as 10 min after LPS stimulation (Figure 3A). IL-6, IL-8 and VEGF expression induced by LPS could be significantly decreased by p38 MAPK inhibitor PD169316, which proved that p38 MAPK pathway was involved in the activation of OSCC cells through TLR4 (*p < 0.05, compared with LPS stimulation alone, Additional file 1:Figure S2). Western blot was also used to analyze the expression of I-κBα in the cell lysates of OSCC cell lines which had been stimulated with LPS. As shown in Figure 3A, I-κBα rapidly degraded within 1 h following stimulation with LPS. Immunofluorescence stain showed that there was a significant increase in nuclear translocation of NF-κB subunit p65 after LPS stimulation in OSCC cell lines (Figure 3B and 3C); the mean fluorescence intensity (MFI) of nuclear NF-κB exhibited a significant increase in OSCC cell lines (Figure 3D), whereas no changes were observed in HIOEC cells (Figure 3B-D). Moreover, we detected increased luciferase activity with the NF-κB luciferase reporter, indicating the enhanced NF-κB activity in CAL-27 cells with LPS stimulation (Figure 3E). These findings suggested that both p38 MAPK and NF-κB signaling pathways were activated by LPS in human OSCC cells.

In order to further identify whether NF-κB and p38 MAPK pathways were activated by LPS through TLR4, we silenced TLR4 using siRNA. Transfection of TLR4 siRNA resulted in 80% reduction of TLR4 protein expression in CAL-27 cells and HB cells (Figure 3F). TLR4 siRNA resulted in partial inhibition of the LPS-induced activation of NF-κB and p38 MAPK pathways compared with the negative control siRNA (NC siRNA). At the same time, a luciferase reporter assay demonstrated that LPS-induced activation of NF-κB was almost completely inhibited by TLR4 siRNA compared with that of the NC siRNA in CAL-27 cells (Figure 3G). These results indicated that LPS-induced activation of NF-κB and p38 MAPK pathways in OSCC cell lines were mediated by TLR4.

### LPS protects OSCC cell lines from cisplatin-induced apoptosis through TLR4

**Figure 4:**
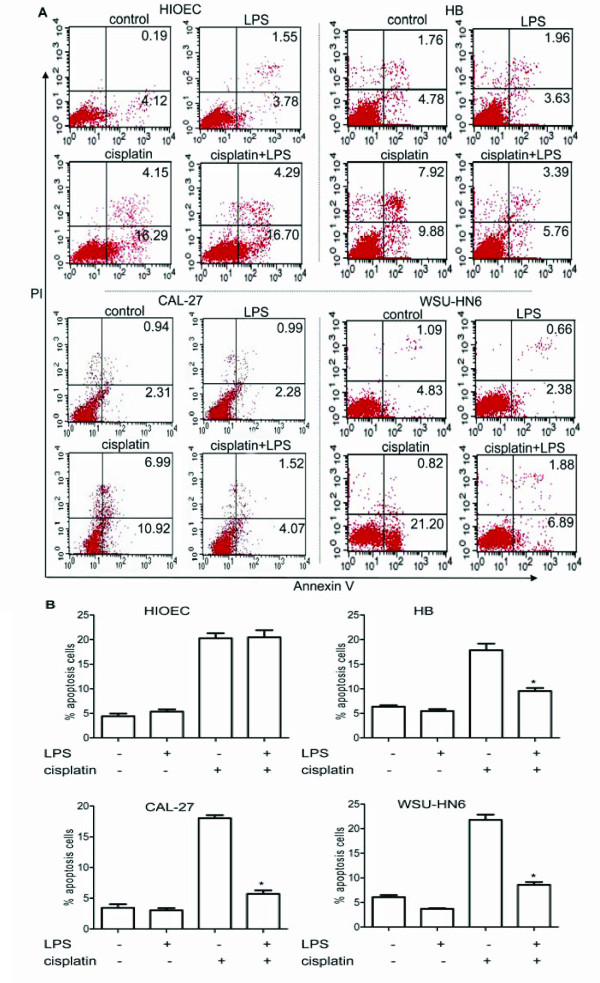
**The effects of LPS pretreatment on the cisplatin-induced apoptosis of HIOEC and human OSCC cell lines. *A. *** HIOEC and OSCC cell lines (5 × 10^5^ ml^-1^) were pretreated with or without 1 μg/ml LPS for 4 h, then stimulated with 1 μg/ml cisplatin for 24 h. The cells were stained with Annexin V and PI and subjected to flow cytometry to determine the percentage of Annexin V^+^PI^-^/Annexin V^+^PI^+^ apoptotic cells. Results are representative of three independent experiments. ***B.*** As depicted, LPS significantly lowered cisplatin-induced apoptosis by elevating the resistance of OSCC cells but not HIOEC cells. (*p < 0.05, compared with the group treated with cisplatin only).

We explored whether LPS could protect OSCC cells from cisplatin-induced apoptosis. OSCC cell lines were pretreated with LPS (1 μg/ml) for 4 h, and then treated with cisplatin for another 24 h. PI and Annexin V staining method was used to examine apoptosis. As shown in Figure 4A, pretreatment of OSCC cells with LPS decreased the cisplatin-induced cells death and apoptosis, resulting in a reduction from 18% to 5.6% in CAL-27 cells, 22% to 8.8% in WSU-HN6 cells and 17.8% to 9.1% in HB cells. However, HIOEC cells showed more susceptibility to cisplatin-induced apoptosis while LPS pretreatment showed no protective effect (Figure 4A and 4B).

**Figure 5:**
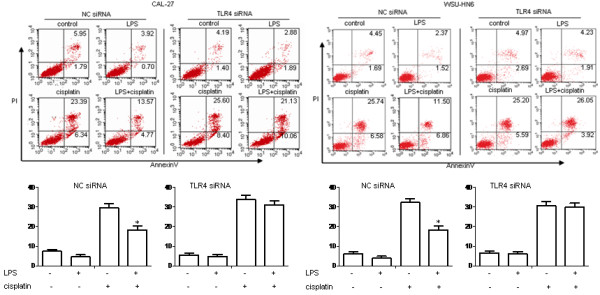
**Effects of TLR4 siRNA on the cisplatin-induced apoptosis in human OSCC cell lines. *A. *** CAL-27 and WSU-HN6 cell lines (5 × 10^5^ ml^-1^) were transfected with NC siRNA or TLR4 siRNA for 48 h, pretreated with or without 1 μg/ml LPS for 4 h, then stimulated with 1 μg/ml cisplatin for another 24 h. The cells were stained with Annexin V and PI and subjected to flow cytometry to determine the percentage of Annexin V^+^PI^-^/Annexin V^+^PI^+^ apoptotic cells. Results are representative of three independent experiments. ***B.*** LPS could not decrease the effects of cisplatin-induced apoptosis in CAL-27 and WSU-HN6 when TLR4 was silenced. (*p < 0.05, compared with the group treated with cisplatin only).

In order to further examine whether the resistance of cisplatin-induced apoptosis upon LPS pretreatment was mediated by TLR4, OSCC cell lines were transfected with TLR4 specific siRNA or a control siRNA (NC siRNA). Reduction of cisplatin-induced apoptosis of OSCC cell lines after LPS pretreatment was abolished when transfected with TLR4 specific siRNA, while NC siRNA could hardly influence cisplatin-induced apoptosis with LPS pretreatment (Figure 5). These results indicated that cisplatin-induced apoptosis decreased by LPS was actually mediated through TLR4 in OSCC cell lines.

## Discussion

We found that TLR4 was expressed in human OSCC cell lines but not on normal human oral epithelial cells. The expression levels of TLR4 and MyD88 *in situ* were correlated with tumor differentiation. TLR4 and MyD88 were highly expressed in well-differentiated and moderately-differentiated tumors, but weakly expressed in poorly differentiated tumors. Cisplatin is widely used for chemotherapy of many malignancies, especially for OSCC. However, the efficiency of cisplatin in the treatment of recurrent tumors is limited because of acquired or intrinsic resistance. In our study, ligation of LPS to TLR4 can protect cisplatin-induced apoptosis in OSCC cell lines but not in HIOEC cells. LPS activated NF-κB and p38 MAPK pathways and triggered target gene transcription. IL-6, IL-8 and VEGF were found to be elevated in OSCC cell lines upon LPS stimulation. Therefore, OSCC cell lines could respond to oral bacteria via the derived LPS, which may lead to apoptotic resistance to cisplatin.

It is well known that IL-6 production and increased circulating level have been emerged as biomarkers of poor prognosis in many human cancers. In ovarian cancer, increased levels of IL-6 in patients’ sera are linked to tumor progression, resistance to apoptosis and chemoresistance [14]. IL-6 is also able to promote tumor angiogenesis and invasion [15-17]. In clinical investigations, high IL-6 levels in the sera of patients with colon carcinoma correlate with tumor size [18]. The effect of IL-6 on hepatocyte proliferation depends on the balance between its pro- and anti-proliferative arms, after the integration of the effects of other transcription factors acting on the same genes as IL-6 [19]. IL-8 is a proangiogenic cytokine/chemokine and anti-apoptotic molecule that can promote tumor metastasis and death resistance. IL-8 and VEGF are involved in the malignant transformation process. It has been reported that IL-8 is overexpressed in ovarian cancer and its level is associated with decreased patient survival and poor clinical outcome [20,21]. VEGF, in addition to inducing angiogenesis, is also an immunosuppressive cytokine that promotes ascite formation through stimulation of vascular permeability [20,22]. The expression of VEGF is negatively correlated with DC numbers in the tumor tissue and peripheral blood of cancer patients [23,24]. Also, VEGF has an inhibitory effect on DC differentiation in patients with non-small-cell lung cancer [25]. Thus, our results show that the elevated production of IL-6, IL-8 and VEGF in OSCC cell lines by LPS stimulation may lead to the development of resistance to cisplatin in human OSCC.

It is well established that NF-κB is an anti-apoptotic transcriptional factor upon tumor cell stimulation with LPS, radiation, and some chemotherapeutic agents. NF-κB has also been previously linked to upregulation of anti-apoptotic protein expression and an increase in cell proliferation [26]. Due to the anti-apoptotic properties of activated NF-κB, its high expression levels in tumor cells are associated with tumor progression and induction of chronic inflammation in the tumor microenvironment [27]. Activation of NF-κB has been shown to induce resistance through the expression of the MDR1 gene [28]. It was also reported that LPS could induce NF-κB activation in colon cancer cells and pancreatic cancer cells [29,30]. Silencing of TLR4 expression in SKOV3 cells resulted in sensitization of SKOV3 cells to PTX-induced apoptosis, and this sensitization was accompanied by the inhibition of cytokines production in response to PTX and LPS [11]. Inhibition of LPS-induced TLR4 signaling could improve therapeutic outcomes by preventing cancer metastasis during the perioperative period of colorectal cancer resection [31]. In our study, OSCC cell lines were shown to be resistant to cisplatin-induced apoptosis upon LPS pretreatment. Moreover, cisplatin-induced apoptosis decreased by LPS was actually mediated through TLR4. TLR4 induced NF-κB pathway activation and nuclear translocation may be associated with the increased transcription of a number of different genes and the production of proinflammatory cytokines such as IL-6, IL-8 and VEGF.

LPS-triggered TLR4 signaling usually activates two different pathways: the NF-κB signal pathway and the p38 MAPK signal pathway. In the cancer cells, Szajnik et al. demonstrated that activation of NF-κB induced by LPS was responsible for the production of cytokines such as IL-6, IL-8 and VEGF in SKOV3 cells [11]. Triggering of TLR4 by LPS induced tumor promotion by the induction of proliferation, activation of NF-κB, p65 binding to DNA, and resistance to NK cell-mediated cytotoxicity accompanied by the increased production of proinflammatory cytokines (IL-6 and IL-8), VEGF [10]. The p38 MAPK pathway has also been shown to be involved in LPS-induced IL-6 secretion in pituitary adenomas and bladder cancer cells [29,32]. However, up to now, the mechanisms by which TLR4 activation with LPS induces resistance to chemotherapy have not been fully understood. One report showed that inhibition of the NF-κB pathway can significantly attenuate LPS-induced apoptosis resistance [9]. In our present study, we found that TLR4 ligation can activate both p38 MAPK and NF-κB pathways in human OSCC cell lines, and inhibition of TLR4 by siRNA can significantly attenuate LPS-induced NF-κB and p38 MAPK activation. We also found that the secretion of cytokines was significantly abrogated by p38 MAPK specific inhibitor, which proved that p38 MAPK was responsible for LPS induced production of IL-6, IL-8 and VEGF in OSCC cells. LPS pretreatment can decrease cisplatin-induced cell death and apoptosis through TLR4 signaling pathway. On the contrary, HIOEC cells, which have very low expression levels of TLR4 and MyD88, did not show these effects. These findings strongly suggest that LPS can provide a survival benefit to OSCC cell lines and alter their sensitivity to cisplatin through activation of p38 MAPK and NF-κB signaling pathway via TLR4.

## Conclusions

In summary, our results indicated that TLR4 was functionally expressed in human OSCC cells and development of resistance to cisplatin in human OSCC might occur through the mechanism involving activation of TLR4 and its signaling pathway. Suppression of TLR4 and its signaling pathway might thus elevate sensitivity to cisplatin and potentially help improve the prognosis of patients with OSCC.

## Methods

### Cell lines and reagents

The human OSCC cell line CAL-27 used in this study was obtained from the American Type Culture Collection (ATCC), which was established in 1982 by J. Gioanni (Centre Antoine Lacassagne, Nice Cedex, France) from tissue taken prior to treatment from a 56 year old Caucasian male with a lesion of the middle of the tongue. CAL-27 cells are epithelial, polygonal with a highly granular cytoplasm. The cell line WSU-HN6, a human OSCC cell line from tongue, was obtained from the National Institutes of Health (NIH). HIOEC and HB cells were obtained from the Laboratory of Oral Oncology, Shanghai Ninth People’s Hospital affiliated to Shanghai Jiao Tong University, School of Medicine [33,34]. HIOEC is human immortalized oral epithelial cells, which had been obtained from normal oral mucosa immortalized by transfection of HPV16 E6/E7 gene. HB cell was establish from the HIOEC by induction with benzo(a)pyrene [34]. HIOEC cells were maintained in defined keratinocyte-SFM (Gibco, NY, USA) medium and other cells were maintained in DMEM supplemented with 10% heat-inactivated fetal bovine serum (Gibco). All cells were cultured in a humidified atmosphere of 5% CO_2_ at 37°C. LPS was obtained from Sigma (MO, USA), and p38 specific inhibitor PD169316 was from Abcam (MA, USA).

### Specimens and immunohistochemistry

Thirty human OSCC specimens were collected from patients who had undergone surgery between 2007 and 2009 in the Department of Oral and Maxillofacial Surgery, Ninth People’s Hospital. All experimental procedures received ethical approval by the Independent Ethics Committee of Shanghai Ninth People’s Hospital affiliated to Shanghai Jiao Tong University, School of Medicine (Number 200926). Pathological characterization of the OSCC patients included in this study is summarized in Table 1. For immunohistochemical examination, OSCC tissues were fixed with 4% paraformaldehyde and embedded with paraffin. Sections of the samples were blocked with 10% goat serum in PBS and incubated overnight at 4°C with either anti-TLR4 antibody (Imgenex, CA, USA) or with anti-MyD88 antibody (Abcam) at a dilution of 1:100. After three washes with PBS, the sections were incubated with peroxidase-conjugated goat anti-mouse/rabbit antibody for 1 h, followed by incubation with 3, 3’-Diaminobenzidine (DAB) substrate for 3 min. Counter-staining was performed with hematoxylin, and then dehydration was performed with ethanol and dimethyl benzene. Slides were mounted with Permount (Santa Cruz, CA, USA) and visualized by Axio Imager (Zeiss, Oberkonchen, Germany). The IHC results in tissues were scored by two independent investigators based on the level of staining intensity as follows: none (−): 0% of stained cells; weak (+): 1-25% of stained cells; moderate (++): 26-50% of stained cells; strong (+++): >50% of stained cells.

### Reverse transcription-PCR (RT-PCR)

To determine TLR expression at the mRNA level, RT-PCR was performed. Tumor cells (2 × 10^6^) were harvested, after cell washing, and total RNA was isolated using Trizol reagent (Invitrogen, CA, USA). RT-PCR was performed according to the manufacturer’s instructions. Reverse transcription was performed with random primers using an Omniscript RT kit (Promega, WI, USA). The sequences of primers for TLR4 were 5’-CAGAGTTGCTTTCAATGGCATC-3’ (forward) and 5’-AGACT GTAATCAAGAACCTGGAGG-3’ (reverse); and 5’-CTCCTCCACATCCCTTCC-3’ (forward) and 5’-CCGCACGTTCAAGAACAGAGA-3’ (reverse) for MyD88. PCR was performed using an Expand High Fidelity PCR System (TaKaRa, Shiga, Japan).

### Flow cytometry

TLR4 expression in cells was evaluated by flow cytometry as follows: cells were collected and then labeled with the APC-labeled mouse anti-human TLR4 antibody (eBioscience, CA, USA) for 30 min at 4°C. The cells were analyzed using Cell Quest Software. Apoptosis was measured by flow cytometry as follows: cells were harvested and washed in PBS, resuspended in pre-diluted binding buffer, and stained with AnnexinV-FITC (BD Biosciences, CA, USA) for 30 min at room temperature. After being washed and resuspended in PI binding buffer, the cells were immediately subjected to apoptosis analyses by flow cytometry using Cell Quest Software.

### Elisa

Cells (5 × 10^5^ ml^-1^) were stimulated with 1 μg/ml LPS for 24 h. Culture supernatants were collected. Cytokines in the supernatants were measured by ELISA (R&D, MN, USA) according to the manufacturer’s instructions.

### Immunofluorescence microscope

Tumor cells deposited on glass slides were washed twice with PBS and fixed in 4% paraformaldehyde in PBS for 20 min. The cells were further permeabilized with 0.1% TritonX in PBS for 8 min, washed and blocked with 5% bovine serum albumin in PBS for 30 min, then treated with monoclonal mouse anti-p65 (Santa Cruz) antibody overnight. FITC-labeled (1:100) anti-mouse IgG served as the secondary antibody. Sections were then mounted in a medium containing Hoechst33342 for 5 min to visualize cell nuclei. Slides were evaluated with a laser scanning confocal microscope TCS SP2 (Leica, Wetzlar, Germany), and Adobe Photoshop 7.0 was used for the digital image analysis.

### Luciferase reporter gene assay used to indicate NF-κB activity

Cal-27 cells were cotransfected with the mixture of 200 ng pNF-κB-Luc and 10 ng pRenilla using the Lipofectamine^TM^ 2000 Reagent (Invitrogen) according to the manufacturer’s instructions. 24 h after transfection, the cells were either left untreated or stimulated with 1 μg/ml of LPS. Cell lysates were assayed for expression of luciferase using a dual luciferase assay kit (Promega). Chemiluminescence, representing the expression of luciferase, was measured in a Junior LB9505 luminometer (Berthold, Wildbad, Germany). Relative luciferase activity (RLA) was obtained by normalizing the luciferase activity with the pRenilla activity. The extent of NF-κB activation was represented by the relative increase in the level of RLA. All transfection experiments were performed in two wells and repeated independently three times. The activity of controls was set at 1.0.

### Western blotting analysis

OSCC cells were stimulated with LPS for various time periods at 37°C and then lysed with M-PER® Mammalian Protein Extraction Reagent. Protein was quantified by the BCA Protein Assay Kit according to the manufacturer’s instruction, separated by SDS-polyacrylamide gel electrophoresis, and then electrotransferred onto a polyvinylidene difluoride membrane. Essential component detection in the cells was performed with the following antibodies via overnight incubation at 4°C: anti-MyD88 (Abcam), anti-I-κBa (Santa Cruz), anti-phosph-p38 (Cell Signaling Technology, MA, USA), and anti-TLR4 (Imgenex). HRP-conjugated secondary Antibody (Pierce Chemical, MO, USA; 1:5000 dilution) was added for 1 h at room temperature, followed by development of reactions in a chemiluminescent detection system. β-actin antibody was used as a control.

### Effects of TLR4-specific siRNA

Expression of TLR4 in the cell lines was temporarily silenced using small interfering RNA (siRNA) of TLR4. The sense and antisense strands of siRNA were: 5-GUCUAGUGGCUAAUUCCUA-3 and 5-UAGGAAUUAGCCACUAGAC-3. Briefly, 2 × 10^5^ tumor cells were seeded in wells of a six-well plate and cultured in DMEM. The next day, the cells were transfected with human TLR4 siRNA (GenePharma Co., Shanghai, China) at different concentrations according to the manufacturer’s instructions. The negative control consisted of siRNA with no homology to known sequences from humans. Cells were incubated in complete DMEM medium at 37°C in an atmosphere of 5% CO_2_. Western blot was used to test TLR4 expression and further experiments were carried out.

### Statistical analysis

Table 1 data were analysed by the statistical software SPSS10.0 for windows (SPSS10.0 Inc., USA). Other statistical analysis was performed using the Student’s *t*-test. Values of *P* <0.05 were considered significant.

## Abbreviations

TLR4: Toll-like receptor 4; LPS: Lipopolysaccharide; OSCC: Oral squamous cell carcinoma; MyD88: Myeloid differentiation primary response gene 88; HIOECs: Human immortalized oral epithelia cells; PAMPs: Pathogen-associated molecular patterns.

## Competing interests

The authors declare that they have no competing interests.

## Author’s contributions

CFX designed the study and interpreted the data and amended the manuscript; CWT provided all the cell lines and gave instructions; SZJ performed the experiments and drafted the manuscript; LQQ participated in immunohistochemistry and flow cytometry analysis; and YDX helped to do the immunofluorescence microscope analysis. All of the authors read and approved the final version of this manuscript.

## Supplementary Material

Additional file 1 **Figure S1.** HB and WSU-HN6 cells (5×10^5^ ml^-1^) were stimulated with LPS (1 μg/ml) for 24 h, and then cytokine and chemokine in the supernatants were assayed using ELISA. **Figure S2.** LPS-induced secretion of cytokines was blocked by the specific p38 MAPK inhibitor PD169316. Click here for file
